# Synchronous bilateral breast cancer with heterogeneous hormone receptor status: a case report

**DOI:** 10.1093/jscr/rjae786

**Published:** 2024-12-12

**Authors:** Prajjwol Luitel, Badal Karki, Sujan Paudel, Asim Shrestha, Suraj Pariyar, Suzita Hirachan

**Affiliations:** Department of General Surgery, Maharajgunj Medical Campus, Institute of Medicine, Tribhuvan University, Kathmandu 44600, Nepal; Department of General Surgery, Tribhuvan University Teaching Hospital, Maharajgunj, Kathmandu 44600, Nepal; Department of General Surgery, Maharajgunj Medical Campus, Institute of Medicine, Tribhuvan University, Kathmandu 44600, Nepal; Department of General Surgery, Maharajgunj Medical Campus, Institute of Medicine, Tribhuvan University, Kathmandu 44600, Nepal; Department of General Surgery, Maharajgunj Medical Campus, Institute of Medicine, Tribhuvan University, Kathmandu 44600, Nepal; Department of General Surgery, Tribhuvan University Teaching Hospital, Maharajgunj, Kathmandu 44600, Nepal

**Keywords:** breast cancer, synchronous bilateral breast cancer, heterogeneous hormone receptor status

## Abstract

The expression of estrogen, progesterone, and HER2 receptors in breast cancer cells helps guide treatment and predict outcomes. When the status of these receptors is heterogeneous, it makes treatment planning more complex. We present the case of a 72-year-old woman with bilateral breast cancer with heterogenous receptor status and subsequent challenges in management.

## Introduction

Breast cancer is one of the most prevalent malignancies worldwide, with ~2.3 million new cases and 685 000 deaths reported in 2020 [1]. It is highly heterogeneous, both clinically and molecularly, and is classified into distinct subtypes based on hormone receptors and HER2 status. Synchronous bilateral breast cancer (SBBC) is defined as the occurrence of cancer in both breasts diagnosed typically within a period of 3 to 6 months [2]. It is a rare presentation, accounting for ~0.2%–2% of breast cancer cases [3]. The heterogeneity is assessed by various modalities including genetic profiling of tumors and assessment of receptor expression status [4].

Hormone receptor status, including ER, PR, and HER2 plays a crucial role in determining prognosis and guiding treatment in breast cancer [5]. The outcome of SBBCs with heterogeneous hormone receptor status can be particularly complex. The presence of different receptor statuses, such as ER-negative in one breast and ER-positive in the other, requires a careful treatment plan, often involving a combination of hormone therapy, chemotherapy, and targeted therapy [6]. The discordance in receptor status between the tumors in each breast often mandates individualized treatment plans and can lead to varying prognoses. Following the SCARE 2023 guidelines, we present a case of SBBC with heterogeneous hormone receptor status, highlighting the management challenges associated with this rare presentation in resource-limited settings [7].

## Case presentation

A 72-year-old Asian female presented with progressively increasing bilateral breast lumps for one month and yellowish-white nipple discharge, redness, and fever for the same duration. She had no pain, weight loss or difficulty breathing and had no history of hypertension, diabetes, or thyroid disorders. She was a former smoker with 5 pack years and alcohol consumer and had ceased these habits 25 years ago. Her medical history included a total abdominal hysterectomy with bilateral salpingo-oophorectomy done 25 years ago, however the reason for it was not identified. She had a history of regular menstrual cycles, with menarche at 14 years and menopause at 53 years, no history of hormonal contraception or radiation exposure. She had no siblings or children, and there was no family history of malignancy.

**Figure 1:**
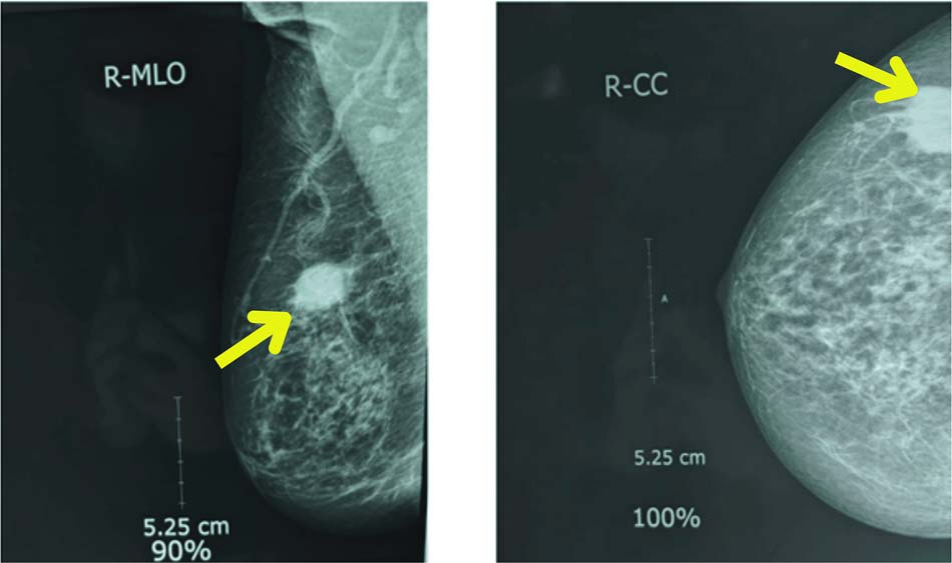
Mammography showing well-defined high-density mass lesions of 3.5 × 3.5 cm with spiculated margins in the upper outer quadrants of right breast.

**Figure 2:**
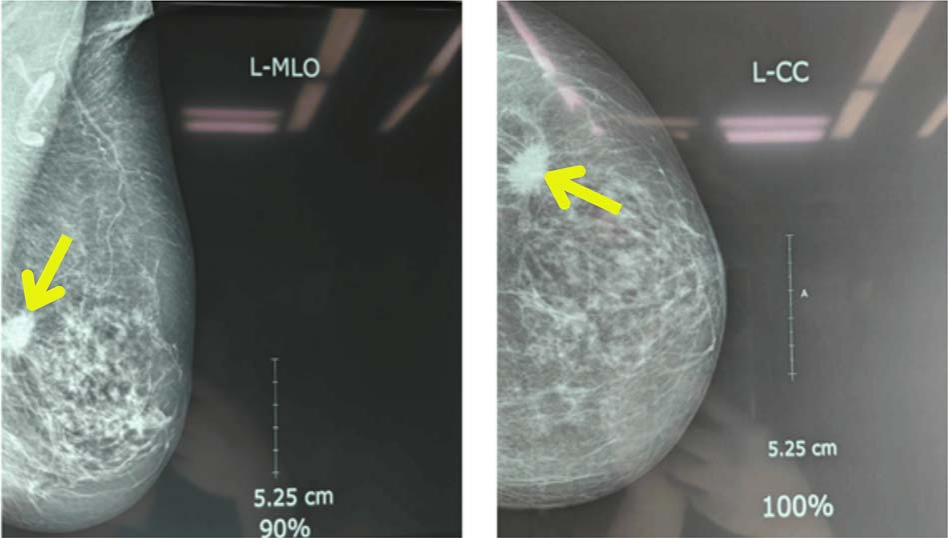
Mammography showing well-defined high-density mass lesions of 3.5 × 3.5 cm with spiculated margins in the upper outer quadrants of left breast.

Upon examination, her general condition was fair and vitals were stable. Local examination revealed 3 cm × 3 cm hard, mobile, painless lumps located at 9 o’clock in the right breast 3 cm from Nipple-areolar complex (NAC) and 4 cm × 3.5 cm similar mass at 3 o’clock in the left breast. The axillary and supraclavicular lymph nodes were not palpable. There was no skin, nipple, or areolar changes. Rest of the systemic examinations were normal. Blood and urine tests were within normal limits. Diagnostic mammography revealed well-defined high-density mass lesions of 3.5 × 3.5 cm with spiculated margins in the upper outer quadrants of both breasts and features suggestive of breast imaging reporting and data system (BIRADS) category 5 (Figs 1 and 2).

**Figure 3:**
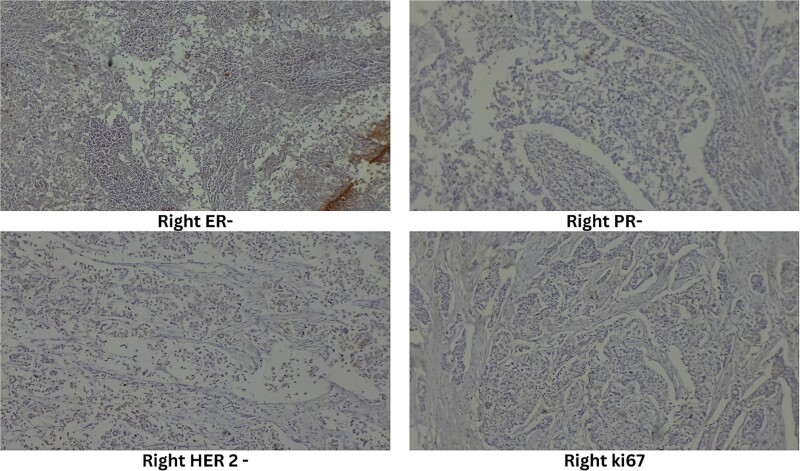
Immunohistochemistry of right breast.

**Figure 4:**
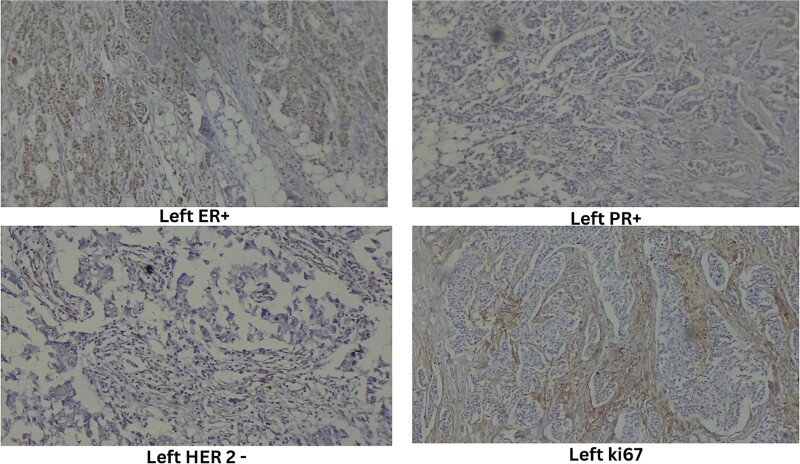
Immunohistochemistry of left breast.

Ultrasound-guided true-cut biopsy confirmed invasive carcinoma of the right breast with a Nottingham histologic score of 8 (Grade 3) and invasive ductal carcinoma of the left breast with a Nottingham histologic score of 5 (Grade 1). Immunohistochemistry of the right breast showed estrogen receptor (ER) negative, progesterone receptor (PR) negative, HER2 negative, and Ki67 immunoreactive in 25% of tumor cell nuclei (Fig. 3). The left breast showed ER positive, PR positive, HER2 equivocal, and Ki67 immunoreactive in 20% of tumor cell nuclei (Fig. 4).

**Figure 5:**
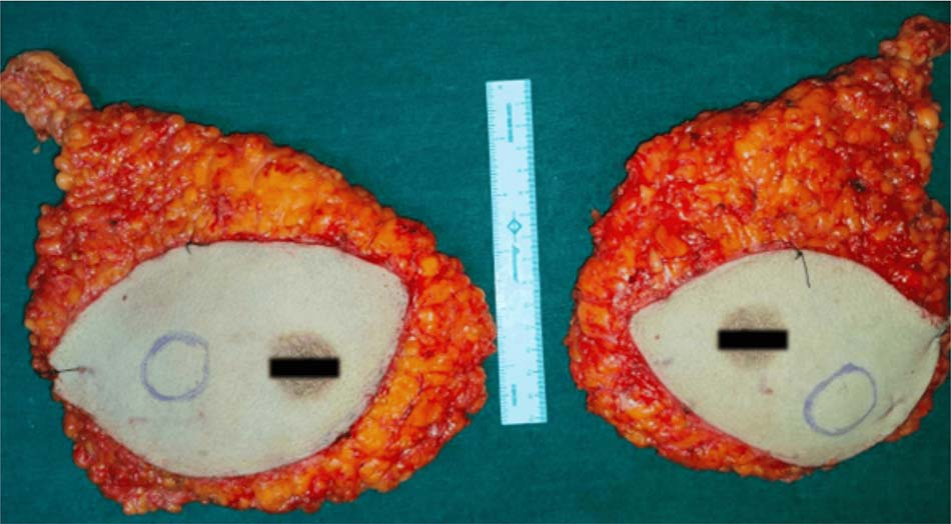
Bilateral MRM specimen.

High-resolution computed tomography of the chest confirmed the lesion without systemic spread. She underwent bilateral modified radical mastectomy (MRM). Intraoperatively, a 3 cm × 3 cm hard, fixed lump was located at 10 o’clock, 3 cm from the NAC in the right breast and a 4 cm × 3 cm hard lump was found fixed at 5 o’clock, 3 cm from the NAC in the left breast. The cut section of the specimen confirmed the above findings (Fig. 5), with no evidence of axillary node metastasis.

The perioperative course was uneventful and she was advised for intravenous adjuvant chemotherapy by the medical oncology team. She denied it and therefore was started on oral capecitabine and letrozole. At a 6-month follow-up, she remained asymptomatic, with no evidence of locoregional recurrence.

## Discussion

Earlier studies have indicated that most secondary synchronous tumors are identified through imaging in patients who initially present with a single breast lesion, and these tumors generally exhibit similar histological characteristics [8]. However, our patient complained of symptoms in both breasts from the initiation. The average age at diagnosis for SBBC patients is 63.1 years, a finding similar to ours [9, 10].

Risk factors for bilaterality include a positive family history of breast cancer, genetic predisposition, lobular type carcinoma, younger age at the diagnosis of the first primary breast cancer, inadequate treatment for the first tumor, and nulliparity [9]. Studies have shown that SBBC can arise from distinct clonal origins, suggesting independent development of each tumor [11]. Our case supports the concept of independent tumorigenesis, given the differing hormone receptor statuses and histologic grades of the tumors in each breast. The patient's previous hysterectomy and bilateral salpingo-oophorectomy could have influenced the hormonal milieu, potentially impacting tumor development and progression.

ER-positive tumors generally benefit from hormone therapy, which can significantly reduce the risk of recurrence and improve survival [6]. On the other hand, ER-negative tumors do not respond to hormone therapy and often require chemotherapy and targeted therapies to achieve optimal outcomes [12].

Traditionally, neoadjuvant therapy has been used to downstage locally advanced or inoperable tumors to enhance surgical outcomes. However, its use in early-stage disease is growing to evaluate tumor response and guide future adjuvant therapies [13]. Additionally, it provides a time window for planning breast reconstruction if a mastectomy is chosen. For patients with triple-negative breast cancer (TNBC) and HER2-positive breast cancer, the response to chemotherapy is a strong indicator of recurrence [14–17]. Thus, the response to neoadjuvant chemotherapy serves as a real-life validation model for predicting the long-term effects of treatment [18]. While the clinical utility of neoadjuvant chemotherapy has been demonstrated, evidence from clinical trials comparing the long-term outcomes of pre- versus post-mastectomy chemotherapy remains insufficient.

However, in this particular case, several patient and clinical factors led to the decision to move straight to bilateral MRM without neoadjuvant therapy. Both the tumors were confined without lymph node or systemic dissemination, and they could be removed without downstaging. Additionally, she had heterogenous receptor status. Given these distinctions, the absence of a clear advantage from downstaging, and the patient’s preference against aggressive chemotherapy, immediate surgical resection was preferred. This approach allowed for tumor removal while respecting the patient’s preferences. The effective resection without any perioperative complications underscores the significance of an individualized approach in managing early-stage bilateral breast cancer, particularly when neoadjuvant therapy does not offer a clear benefit compared to immediate surgical treatment.

## Conclusion

The heterogeneity in receptor status significantly complicates the treatment of SBBC. The tumor biologics along with patient autonomy should be considered during decision-making in cancer treatment. Each tumor must be addressed as a separate condition, with the treatment strategy specifically designed to maximize the likelihood of achieving a complete pathological response in both tumors and enhancing long-term outcomes.

## Declaration

All the authors declare that the information provided here is accurate to the best of our knowledge.
